# Gaps and challenges in the management of atrial fibrillation in the Philippines

**DOI:** 10.1016/j.amsu.2022.104586

**Published:** 2022-09-07

**Authors:** Jeremy A. Ceriales, Leslie Faye T. Cando, Maria Llaine J. Callanta, Ourlad Alzeus G. Tantengco

**Affiliations:** College of Medicine, University of the Philippines Manila, Manila, Philippines; Department of Biochemistry and Molecular Biology, College of Medicine, University of the Philippines Manila, Manila, Philippines; College of Medicine, University of the Philippines Manila, Manila, Philippines

Atrial fibrillation (AF) is the most common sustained cardiac arrhythmia. Chen and colleagues reported a 0.88% prevalence of AF in Shanghai, China. The associated risk factors include older age, female sex (>80 years), male sex (<80 years), and cardiovascular diseases [1]. The prevalence of AF is predicted to increase due to the increasing aging population and social industrialization, especially in developing countries. In this study, we share the epidemiology of AF in the Philippines, a developing country in Southeast Asia.

Even though AF is the most common sustained arrhythmia, data on AF in the Philippines remains scarce [2]. The prevalence of AF in SEA is estimated to range from 2.6 to 23.04% [3]. Projections from available prevalence data predict that Asia will see a rapid increase in AF burden to an estimated 8.3 million by 2050. Structural heart diseases such as heart failure and valvular heart disease, especially rheumatic heart disease in developing countries, are common underlying causes of AF in Asia. Hyperthyroidism, CKD, obesity, OSA, diabetes, and hypertension also increase the risk of AF in Asians [2].

As AF substantially increases the occurrence of stroke by 4 to 5-fold, it is of utmost importance to adhere to a therapeutic plan. Management of patients is currently patterned to Western practice guidelines. Recommendations from the 10.13039/501100000860European Society of Cardiology (ESC) promote an integrated therapeutic plan for AF, which include rhythm and rate control, stroke prevention, and management of comorbidities through lifestyle modifications, healthcare/patient education, and psychosocial support [4]. ESC recommends oral anticoagulants (VKAs or NOACs) to patients with risk factors for stroke by CHA2DS2-VASc score with INR monitoring. The use of antiplatelet is not highly recommended for stroke prevention in AF [5].

AF causes a significant burden to patients, physicians, and healthcare systems globally [4]. Management of the risk factors of AF is of prime importance in addressing the burden of AF in the Philippines. Management is multidisciplinary and multifaceted—lifestyle modifications, pharmacologic, and non-pharmacologic interventions. Screening and interventions should be more accessible and affordable since risk factors for AF are easily recognizable and manageable. More local research on AF should be done to raise public awareness and forward health programs. Guidelines should be personalized to Filipinos through research and collaborative efforts. To address these gaps and challenges, we call on the government to increase its attention and fund allocation to the health and research sectors in the Philippines.

## Ethical approval

Not applicable.

## Please state any sources of funding for your research

This study did not receive any funding.

## Author contribution

All authors contributed equally in writing this manuscript.

## Please state any conflicts of interest

We declare no competing interests.

## Consent

Not applicable.

## Registration of research studies

1. Name of the registry: Not applicable
2. Unique Identifying number or registration ID: Not applicable
3. Hyperlink to your specific registration (must be publicly accessible and will be checked): Not applicable

## Guarantor

Jeremy A. Ceriales, BSc, Leslie Faye T. Cando, BSc, Maria Llaine J. Callanta, MD, PhD, MPM, *Ourlad Alzeus G. Tantengco, MD, PhD.
